# Spatial effect of carbon neutrality target on high-quality economic development—Channel analysis based on total factor productivity

**DOI:** 10.1371/journal.pone.0295426

**Published:** 2024-01-24

**Authors:** Yiniu Cui, Cheng Zhong, Jianhong Cao, Mengyao Guo, Meng Zhang

**Affiliations:** 1 School of Economics, Yunnan University, Kunming, China; 2 School of Business, Pingxiang University, Pingxiang, China; 3 Yuquan Institute, University of Chinese Academy of Sciences, Beijing, China; 4 School of Business, Guangxi University, Nanning, Guangxi, China; Sichuan Agricultural University, CHINA

## Abstract

This study utilizes panel data from 30 provinces in mainland China from 2011 to 2020 to investigate the impact of carbon-neutral development on economic high-quality development by constructing an economic high-quality development index and a carbon-neutral development index. Firstly, the study examines the effects of carbon-neutral development on economic high-quality development using baseline regression and spatial Durbin regression. The results indicate that carbon-neutral development has a positive direct effect on economic high-quality growth, but there are negative spatial spillover effects. Secondly, this study employs total factor productivity (TFP) as an intermediate variable in the mediation model regression. The findings demonstrate that carbon-neutral development significantly improves TFP, and the significant improvement in TFP promotes high-quality economic growth. Lastly, the study conducts regional heterogeneity analysis and finds a significant promoting effect of carbon-neutral development on economic high-quality development in the eastern and central regions of China, while it is not significant in the western region. Therefore, it is recommended that China, in the process of achieving carbon-neutral growth, consider the geographical connections between different regions to prevent negative spillover effects. Additionally, regional heterogeneity should be taken into account when formulating relevant policies to promote economic high-quality development.

## 1. Introduction

China’s economy is currently undergoing a crucial transformation from rapid expansion to high-quality development. The 20th National Congress report emphasizes the primary objective of building a comprehensive socialist modern nation through high-quality development. To achieve this, it is necessary to focus on promoting high-quality development by integrating strategies to boost domestic demand and deepen structural reforms on the supply side. This approach will enhance the endogenous momentum and reliability of the domestic cycle, elevate the quality and level of the international cycle, expedite the development of a modern economic system, prioritize the increase in total factor productivity (TFP), and emphasize resilience. China’s rise as the world’s second-largest economy has attracted global attention due to its high growth rates. However, the pursuit of high-quality economic development still faces challenges such as low industrial efficiency, significant pollution issues in energy-intensive industries, inadequate capacity for independent innovation, trade growth driven by quantity rather than quality, and uneven improvement in people’s living standards [1]. This signifies a departure from China’s previous growth model, which prioritized the rate of economic growth. In the future, economic growth will be centered around the quality rather than the quantity of growth [2]. To achieve the organic integration of quantity and quality in economic development, new drivers of economic growth beyond capital, labor, and resources need to be sought [3]. High-quality development is characterized by higher quality, greater efficiency, greater equity, and greater sustainability. It involves modifying the economic development mode, optimizing the economic structure, and transforming the growth impetus. The primary objectives are to enhance TFP and construct a modern economic system [4]. High-quality development is multifaceted, systematic, dynamic, and long-term in nature, encompassing economic, ecological, and social aspects. In light of the evolving domestic and international development environment, China’s growth path and strategy for the new era emphasize high-quality economic development. This is characterized by a high-quality and efficient supply system, increased efficiency, sustainability, stability, security, and a higher level of openness to the world [5, 6].

In recent years, China has made significant progress towards becoming a modernized socialist state and has embarked on a path of high-quality economic development. However, this progress has come at a cost, with extensive energy consumption and excessive greenhouse gas emissions. In light of the pressing global concern of global warming, the international community expects China to make more commitments and take action to reduce carbon emissions [7, 8]. During the 75th session of the United Nations General Assembly’s general debate on September 22, 2020, China declared its commitment to reducing carbon emissions. It announced its intention to strive for a peak in carbon dioxide emissions by 2030 and work towards achieving carbon neutrality by 2060. This demonstrates China’s determination to address the issue of carbon emissions. The 20th Report emphasizes the strategic task of establishing China’s ecological civilization in the new era. It elevates "modernization in harmony with nature" as one of the core aspects of "Chinese modernization." The report promotes green development and the coexistence of humanity and nature. It emphasizes the importance of integrated protection and systematic management of natural resources, including mountains, water, forests, lakes, grasslands, and deserts. The report also highlights the need for coordinated efforts in industrial restructuring, pollution control, and ecological protection. To achieve the goals of carbon emissions reduction, pollution control, and green expansion, China aims to establish new technologies, industries, investments, transportation, buildings, and energy systems. The country encourages innovation and the application of green and low-carbon technologies. It provides increased support for green industries and establishes a market-based climate investment and financing framework. These initiatives are aimed at effectively controlling carbon dioxide emissions and facilitating a fundamental shift in the economic model from a focus on quantity to one on quality. It also involves transitioning from heavy industries to lighter and more sustainable industries [9].

When examining the relationship between carbon neutrality and high-quality economic development, it is essential to consider the role of total factor productivity (TFP). TFP is a measure of economic efficiency and productivity, reflecting the level of output an economic system can generate given a set of inputs. Improving TFP means achieving higher output with the same resource utilization, leading to efficient and high-quality economic development [10]. TFP plays a significant role in the connection between carbon neutrality and high-quality economic development. Carbon neutrality can drive improvements in TFP by stimulating technological innovation and industrial upgrading, thereby promoting the transition to low-carbon development. These efforts can enhance resource and energy efficiency, thereby increasing TFP. For example, by adopting clean energy as a substitute for traditional energy sources, companies can reduce energy consumption and carbon emissions, improving production efficiency and TFP. Furthermore, enhancing TFP can facilitate high-quality economic development. Improving TFP means achieving higher output with the same resource input, which leads to greater efficiency and competitiveness in the economy. Enterprises and industries with high TFP are better equipped to meet market demands, provide higher-quality products and services, and gain larger market shares and competitive advantages. Additionally, improving TFP can drive optimization and upgrading of the industrial structure, fostering sustainable economic development. Therefore, carbon neutrality can promote improvements in TFP, and an enhancement in TFP can facilitate high-quality economic development. This creates a virtuous cycle between carbon neutrality and high-quality economic development. By continuously improving TFP, the economy can utilize resources more efficiently, reduce carbon emissions and environmental pressures, and achieve carbon neutrality goals. At the same time, the promotion of carbon neutrality drives technological innovation and structural adjustments in enterprises and industries, improving TFP and propelling high-quality economic development [11].

The academic research contributions of this article are as follows: Firstly, this article builds a comprehensive index that goes beyond the traditional focus on studying carbon emissions and carbon sequestration in isolation. This index transforms the measurement of carbon neutrality development from a single carbon emission reduction indicator to a multidimensional and comprehensive dynamic indicator. As a result, it enables a more comprehensive study of the impact of carbon neutrality goals on high-quality economic development. This comprehensive index provides a framework for evaluating the progress of carbon neutrality and its integration with high-quality economic development. Secondly, we explore the mediating role of total factor productivity in the relationship between carbon neutrality development and high-quality economic development. Traditional studies on the Environmental Kuznets Curve typically focus on the direct relationship between environmental pollution and economic development. In this study, we consider total factor productivity as a mediating variable to investigate the impact mechanism of carbon neutrality development on high-quality economic development. This consideration allows for a more comprehensive analysis of the relationship between carbon neutrality development and economic development. It helps us understand the impact of carbon neutrality on economic growth and innovation, as well as how to address economic challenges in carbon neutrality development by improving total factor productivity. Finally, this paper investigates the impact of carbon neutrality development on high-quality economic development through regional heterogeneity. Traditional research often considers the entire country or region as a whole when examining the relationship between carbon neutrality development and high-quality economic development [12]. By studying different regions, we can gain a better understanding of the impact of carbon neutrality on high-quality economic development and reveal the interactions among multiple factors influencing the relationship. Through the analysis of regional heterogeneity, we can identify the differences in carbon neutrality development and high-quality economic development across various regions, providing a basis for formulating regionally tailored policies.

## 2. Literature review

### 2.1 Carbon neutralization and high-quality economic development

Scholars have reached a general consensus that high-quality economic development requires not only a focus on economic stability but also attention to environmental sustainability. Carbon neutrality development operates within the framework of environmental sustainability, emphasizing sustainable economic growth through reducing carbon emissions and environmental pollution. According to the theory of environmental sustainability, economic activities should operate within the substitutability of resources and the capacity of natural systems to ensure ecological balance and the long-term sustainable use of resources. The establishment and achievement of carbon neutrality goals can effectively address the ecological and environmental management challenges brought by China’s early rapid economic growth, thus promoting high-quality economic growth. When studying the impact of carbon neutrality development on high-quality economic development, the Environmental Kuznets Curve (EKC) can be used to explore the relationship between carbon neutrality and high-quality economic development. The EKC suggests that in the early stages of economic development, with the acceleration of industrialization and urbanization, economic activities lead to an increase in environmental pollution [13, 14]. During this stage, due to the significant increase in economic activities, carbon emissions continue to rise, causing severe environmental pollution alongside rapid economic development. However, once the level of economic development reaches a certain stage, people begin to pay attention to environmental sustainability and take relevant measures to reduce environmental pollution. At this point, people gradually realize that the environment is a key driving factor for economic soft power, and promoting carbon reduction is beneficial for improving the livability and quality of life of residents, as well as achieving the goal of "improving livelihood" within the context of high-quality economic development. Based on the Porter hypothesis, proactive carbon reduction can stimulate the optimization of low-carbon technologies and economic structures, thereby achieving the goal of "improving quality and efficiency" in high-quality economic development [15]. Under the concept of carbon neutrality, as the idea of harmonious coexistence between humans and nature becomes deeply rooted, people’s consumption patterns will change, leading to adjustments in local energy industry structures and consumption patterns. Achieving "sustainability" in high-quality economic development involves promoting green consumption, encouraging the development of green low-carbon production and lifestyles, and reducing waste generation and emissions [16, 17].

This paper presents hypothesis 1(a): Carbon neutral development will drive high-quality economic development.

In addition, carbon neutrality has externalities, which means it will have an impact on other regions while promoting local economic development. The theory of externalities refers to the positive or negative effects of economic production or consumption activities on third parties that are not directly involved and cannot be internalized through market transactions. Externalities can be production externalities, which arise from the production activities’ impact on others, or consumption externalities, which arise from the consumption activities’ impact on others. In the context of carbon neutrality development, reducing carbon emissions can be seen as a corrective measure for externalities. This impact is mainly reflected in the transition of an area away from fossil fuels and towards clean energy and low-carbon technologies, which can also influence adjacent areas to undertake technological transformations and encourage other regions to undergo green transformations. However, during the initial implementation of carbon neutrality policies in an area, some enterprises and individuals may transfer their carbon production activities to other areas, thereby causing new pollution in those areas. At the same time, the implementation of carbon neutrality policies will inevitably force high-polluting enterprises to undertake technological transformations, which will implicitly increase business costs. However, the companies or individuals that emit carbon cannot fully bear the costs of these policies, and these issues cannot be resolved through market transactions. This may lead to negative spillover effects of carbon-neutral development on high-quality economic development. Based on the externalities impact of carbon neutrality, further exploration of the impact of carbon neutrality on other regions is needed when discussing its economic impact on the local region.

This paper presents hypothesis 1(b): Carbon-neutral development may have negative spatial spillover effects on high-quality economic development.

### 2.2 Carbon neutrality and total factor productivity

Scholars generally believe that in order to achieve carbon neutrality goals, pilot cities implementing low-carbon policies often introduce a series of preferential policies such as fiscal support, tax reduction, financial subsidies, and talent incentives to encourage enterprises and individuals to improve production methods and business models. In this process, these preferential policies effectively reduce production costs and technological improvement pressures for enterprises, providing strong support for improving total factor productivity [18, 19]. The development of carbon neutrality requires the adoption of new clean and low-carbon technologies to reduce carbon emissions and environmental pollution. The theory of technological progress suggests that technological advancements can improve production efficiency and total factor productivity. Policies and market demand related to carbon neutrality development will promote the research and application of green technologies, driving technological progress and innovation, thereby improving production efficiency and total factor productivity [20]. In this context, the core of carbon neutrality promoting total factor development lies in technological innovation [21, 22]. They believe that technological innovation and improved resource allocation efficiency are the main ways to enhance total factor productivity, and carbon neutrality development provides a solid foundation for pursuing these avenues. However, some scholars argue that the development of carbon neutrality may hinder the growth of total factor productivity, primarily due to the consequences of establishing carbon trading mechanisms within the framework of "carbon neutrality" [23, 24]. Carbon emissions trading aims to internalize the external costs of carbon emissions through price mechanisms, thereby reducing enterprises’ CO2 emissions [25]. However, due to cost-driven effects and income incentives, enterprises must either purchase carbon emission quotas or reduce production to keep their carbon emissions within the established quota range. However, regardless of the approach taken, it will reduce enterprise profits, which contradicts the goal of profit maximization. Therefore, enterprises can implement measures such as technological innovation and optimization of factor allocation to improve total factor productivity.

This paper presents hypothesis 2: Carbon neutral development will enhance total factor productivity.

### 2.3 Total factor productivity and high-quality economic development

In the context of carbon neutrality, total factor productivity (TFP) serves as a key indicator to measure economic efficiency. Its improvement will contribute to achieving high-quality economic development and the transformation towards green development. Therefore, the rational and efficient use of energy, the establishment of low-carbon production and lifestyle, and the maximization of TFP are necessary conditions for achieving high-quality economic development [26]. Scholars generally believe that the promotion of TFP for high-quality economic development mainly relies on the following two aspects. Firstly, the development of TFP will promote industrial transformation and upgrading, enabling the country to carry out deeper reforms in industrial structure and mode, and achieve high-quality economic development [27, 28]. The improvement of TFP will encourage the improvement of production technology in various sectors, which will encourage the transformation of industrial structure from low value-added traditional industries to high value-added high-tech and strategic new industries, thus promoting economic growth [29]. The neoclassical growth theory suggests that technological progress is the main driving force for economic growth. The improvement of TFP is closely related to technological innovation and progress. By introducing new production technologies, improving production processes, and increasing TFP, high-quality economic development can be promoted. Additionally, effective market mechanisms will facilitate the promotion of TFP for high-quality economic development. Institutional economics theory suggests that a sound institutional environment is crucial for economic development. Strengthening TFP requires a favorable institutional environment to promote innovation, technological progress, and efficient resource allocation. By establishing sound market mechanisms, ensuring sufficient protection of intellectual property rights, and promoting a fair competitive environment, the economy can enhance TFP and achieve high-quality economic development. With the promotion of effective market mechanisms, dynamic micro entities, and moderate macro-control, the process of local marketization can be accelerated, energy and pollution-intensive and inefficient enterprises can be eliminated, and ecological capital can be improved. These factors contribute to the increase in TFP and encourage high-quality economic growth [30, 31].

This paper presents hypothesis 3: Total factor productivity can drive high-quality economic development.

### 2.4 Literature review summary

This study aims to address the research gap in the existing literature by constructing a Carbon Neutrality Development Index that comprehensively reflects the relationship between carbon neutrality development and high-quality economic development. While previous studies have primarily focused on the singular consideration of the relationship between carbon emissions, carbon sequestration, and the economy, this study fills the void by providing a more holistic perspective. In addition, this study expands the research path on the relationship between carbon neutrality development and high-quality economic development by incorporating Total Factor Productivity (TFP) as a key mediating variable. This approach enriches the research findings and provides a more comprehensive understanding of the complex relationship between carbon neutrality development and high-quality economic development. Furthermore, this study contributes to the existing literature by reviewing and drawing insights from previous research. It offers valuable insights into achieving a balance between carbon neutrality and sustainable economic growth. By considering various factors and adopting a comprehensive approach, this study sheds light on the potential pathways to achieve both carbon neutrality and high-quality economic growth. Overall, this study contributes to the academic discourse by providing a comprehensive analysis of the relationship between carbon neutrality development and high-quality economic development. It fills the research gap by considering multiple dimensions and incorporating TFP as a mediating variable. The study also draws upon existing literature, providing valuable insights for achieving a balanced approach between carbon neutrality and high-quality economic growth.

## 3. Model construction and data

### 3.1 Model construction

#### 3.1.1 OLS panel regression model construction

To verify the above assumptions, this paper first constructs a basic linear model (OLS) to study the impact of carbon neutral development on high-quality economic development. The model is shown in Formula (1):

$${ggdpsq}_{it}=a_{0}+\beta_{1}{score}_{it}+\beta_{2}{eri}_{it}+\beta_{3}{open}_{it}+\beta_{4}{ur}_{it}+\beta_{5}{lnfdi}_{it}+\beta_{6}{lnpm}_{it}+\beta_{7}gap++{ℇ}_{it}$$ (1)

In Formula (1), *i* represents the region and *t* represents the year; *score*_*it*_ is the core explanatory variable, representing the level of carbon neutral development; *ggdpsq*_*it*_ is the core explained variable, representing the level of high-quality economic development; *eri* indicates the intensity of environmental regulation; *open* represents the degree of openness; *ur* stands for registered unemployment rate; *lnfdi* represents foreign direct investment; *lnpm* represents PM2.5 concentration; *gap* represents the urban-rural income gap; *β*_1_~*β*_7_ represents variable coefficient; *a*_0_ represents a constant term; *ε* is a random error term.

#### 3.1.2 Construction of spatial Durbin model

To explore the impact of carbon neutral development on high-quality economic development in space, this paper constructs a spatial Durbin model (SDM), as shown in Formula (2):

$${ggdpsq}_{it}=\rho \sum_{j=1}^{n}\;W_{ij}\;{ggdpsq}_{it}+{\beta_{1}score}_{it}+\theta_{1}\sum_{j=1}^{n}\;W_{ij}\;{score}_{it}+\lambda X_{it}+\mu_{i}+\lambda_{t}+\varepsilon_{it}$$ (2)

In Eq (2), *i* represents area, *t* denotes year; *W* is *n*×*n* order geographical distance spatial weight matrix; *ρ* is the spatial autocorrelation coefficient of the explained variable to measure the possible spatial correlation of the explained variable between regions; *β* is the regression coefficient of explanatory variables, which measures the influence of explanatory variables on explained variables in the region; *θ* is the spatial regression coefficient of explanatory variables to measure the spatial spillover effect of explanatory variables; *X*_*it*_ denotes control variables, includ*e eri*; *open*; *ur*; *lnfdi*; *lnpm*; *gap*.*μ*_*i*_ is the spatial fixed effect, *λ*_*t*_ is the time fixed effect, *ε*_*it*_ is a random error term.

Choosing a geographic weight matrix is necessary to consider the spatial factors in the relationship between carbon neutrality development and high-quality economic development. Here are the reasons for selecting a geographic weight matrix: (1) Spatial Interactions: Geographic location may have spatial interactions that influence the relationship between carbon neutrality development and high-quality economic development. Adjacent regions may experience resource flows, technology transfer, economic connections, and other factors that can impact both aspects. A geographic weight matrix captures these spatial interactions and helps analyze the influence of geographical location on the relationship; (2) Spatial Heterogeneity: Different regions exhibit variations in natural environment, economic structure, and development levels, which can affect the relationship between carbon neutrality development and high-quality economic development. By considering factors such as geographical distance, topographic features, and transportation networks, a geographic weight matrix quantifies the spatial heterogeneity among regions, enabling a more accurate analysis of the relationship; (3) Spatial Dependence: Geographical location can create spatial dependence in the relationship between carbon neutrality development and high-quality economic development. The development level of carbon neutrality and economic quality in one region may be influenced by neighboring regions. A geographic weight matrix helps identify and quantify this spatial dependence, facilitating a better understanding of the relationship.

By incorporating a geographic weight matrix, researchers can account for the influence of geographic factors when analyzing the relationship between carbon neutrality development and high-quality economic development. This approach provides more comprehensive and accurate research outcomes, deepening our understanding of the spatial relationship between the two aspects and offering a scientific basis for policymaking and measures.

#### 3.1.3 Construction of mediating effect model

To investigate what role total factor productivity (*tfp*) plays and what role it plays in the mechanism of the impact of carbon neutral development on high quality economic development, a mediating effect model is constructed in this paper, as follows:

$$ggdpsq=a_{0}+\beta_{1}{score}_{it}+\beta_{2}{eri}_{it}+\beta_{3}{open}_{it}+\beta_{4}{ur}_{it}+\beta_{5}{lnfdi}_{it}+\beta_{6}{lnpm}_{it}+\beta_{7}gap++{ℇ}_{it}$$ (3)

$${tfp}_{it}=a_{0}+\beta_{1}{score}_{it}+\beta_{2}{eri}_{it}+\beta_{3}{open}_{it}+\beta_{4}{ur}_{it}+\beta_{5}{lnfdi}_{it}+\beta_{6}{lnpm}_{it}+\beta_{7}gap++{ℇ}_{it}$$ (4)

$${ggdpsq}_{it}=a_{0}+\beta_{1}{score}_{it}+\beta_{2}{tfp}_{it}+\beta_{3}{eri}_{it}+\beta_{4}{open}_{it}+\beta_{5}{ur}_{it}+\beta_{6}{lnfdi}_{it}+\beta_{7}{lnpm}_{it}+\beta_{8}gap+{ℇ}_{it}$$ (5)

In Formulas (3)–(5), *i* represents the region and *t* represents the year; *score*_*it*_ is the core explained variable, representing the level of carbon neutral development; *ggdpsq*_*it*_ is the core explanatory variable, representing the level of high-quality economic development; *tfp* is an intermediate variable, representing total factor productivity. Control variables include *eri*; *open*; *ur*; *lnfdi*; *lnpm*; *gap*; *β*_1_−*β*_8_ represents variable coefficient; *a*_0_ represents a constant term; *ε* is a random error term.

### 3.2 Variable description and data source

#### 3.2.1 Explained variable

This paper uses Feng et al. (2020) to construct a high-quality economic development system from five dimensions: innovative development, coordinated development, green development, open development, and shared development [32]. Among them, innovative development are represented by indicators such as R&D investment intensity, investment efficiency and technology transaction activity that can represent innovation and development; Coordinated development adopts urban and rural structure, demand structure, industrial structure and other indicators that can reflect the structure of economic development to replace; The degree of green development is measured by the elastic coefficient of energy consumption, wastewater per unit GDP output, waste per unit GDP output and the government’s ecological concern; Open development is expressed by the degree of finance and marketization; Shared development is represented by indicators such as the proportion of workers’ remuneration, the growth elasticity of residents’ income, and the Thiel index to measure the fairness of development. The specific indicators are shown in Table 1:

**Table 1:** High-quality economic development system.

Level 1 indicators	Level 2 indicators	Level 3 indicators	Code	Symbols	Indicator Description
High-quality economic development indicators	Innovative Development	R&D investment intensity	X1	+	R&D investment of enterprises above designated size/regional GDP
		Investment efficiency	X2	-	Investment rate/GDP growth rate
		Technology Trading Activity	X3	+	Technical transaction volume/regional GDP
		Number of patent applications	X4	+	Total number of patents applied by regions
	Coordinated Development	Demand structure	X5	+	Total retail sales of social consumer goods/regional GDP
		Urban and Rural Structure	X6	+	Urban population/total population
		Industry Structure	X7	+	GDP/total GDP of the tertiary industry
		Government Debt Pressure	X8	-	Local government debt/regional GDP
	Green Development	Energy consumption elasticity coefficient	X9	-	Energy consumption growth rate/GDP growth rate
		Wastewater per unit output	X10	-	Total wastewater discharge/regional GDP
		Exhaust gas per unit output	X11	-	Sulfur dioxide emissions/regional GDP
		Government ecological concern	X12	+	Local government issues ecological attention document
	Open Development	Marketization degree	X13	+	Degree of marketization by region
		Openness	X14	-	Total import and export/regional GDP
		Development degree of financial institutions	X15	+	Total deposits and loans of financial institutions/regional GDP
	Shared Development	Proportion of labor remuneration	X16	+	Labor remuneration/regional GDP
		Residents’ income growth elasticity	X17	+	Per capita disposable income growth rate of residents/regional GDP growth rate
		Urban-rural consumption gap	X18	-	Total consumption of urban residents—total consumption of rural residents
		Proportion of financial expenditure for people’s livelihood	X19	+	Local government expenditure on education, medical care, social security, employment, etc

#### 3.2.2 Core explanatory variable

The term "carbon neutrality" refers to the total amount of carbon dioxide emissions that a nation, business, product, activity, or individual produces over the course of a specific time period, whether directly or indirectly. The nation’s carbon dioxide emissions can be balanced to achieve positive and negative offsets and relative "zero" emissions through afforestation, energy conservation, emission reduction, and other methods. Based on the basic concept of carbon neutrality, this paper selects two categories of indicators with reference to Zhang et al., (2021), Bai et al., (2023), Wei et al., (2022) and Xu et al., (2022) [33–36]. One is conducive to increasing carbon dioxide emissions, and the other is conducive to curbing carbon dioxide emissions. These indicators are allocated and divided into six categories: government ambition, infrastructure, natural endowment, industrial dimensions, energy consumption, and economy and science and technology [36, 37]. The government’s ambition represents the government’s determination and action to achieve the goal of carbon neutrality, which is specifically reflected in the policy documents and relevant pilot policies issued by the government. Therefore, ecological attention, green development attention, low carbon policy pilot policies (using dummy variables, low carbon pilot cities are 1, and non-low carbon pilot cities are 0), and carbon exchanges (using dummy variables, carbon exchanges are 1, and no carbon exchanges are 0) are selected; The infrastructure details include the indicators that mainly affect the carbon neutral target in human activities, including: heating area, urban lighting, public vehicles, private vehicles, green land, construction land, etc.; The natural endowment is mainly expressed by the index of forest area, lakes, reservoirs and other natural factors that will affect carbon neutralization; The industrial dimension is mainly measured from the perspective of the proportion of secondary and tertiary industries, agriculture, animal husbandry, etc.; Energy consumption is measured by consumption of coal, natural gas, oil, etc.; The economy and technology are mainly measured by green finance, green patents and other indicators closely related to low-carbon development. Specific indicator selection and related contents are shown in Table 2:

**Table 2:** Carbon neutral development index system.

Level 1 indicators	Level 2 indicators	Level 3 indicators	Code	Symbol	Source
Carbon neutral development index	Government ambition	Ecological Attention	X1	+	Government documents at all levels
		Green development attention	X2	+	Government documents at all levels
		Digital attention	X3	+	Government documents at all levels
		Pilot policy of carbon exchange	X4	+	National Development and Reform Commission Work on Pilot Carbon Emission Trading
		Low carbon policy pilot	X5	+	Notice of National Development and Reform Commission on Carrying out the Pilot Work of Low Carbon Provinces and Cities, and Notice on Organizing the Recommendation and Application of the Second Batch of Low Carbon Pilot Provinces and Cities
	Infrastructure Details	Heating area	X6	-	China Statistical Yearbook
		Urban road lighting	X7	-	China Statistical Yearbook
		Number of bus and trolley bus operations	X8	+	China Statistical Yearbook
		Taxi	X9	+	China Statistical Yearbook
		Private car ownership	X10	-	China Statistical Yearbook
		Civil automobile ownership	X11	-	China Statistical Yearbook
		Urban green area	X12	+	China Statistical Yearbook
		Park green area	X13	+	China Statistical Yearbook
		Area of urban construction land	X14	-	China Statistical Yearbook
	Natural endowment	Forest coverage	X15	+	China Statistical Yearbook
		Forest land area	X16	+	China Statistical Yearbook
		Plantation area	X17	+	China Statistical Yearbook
		Number of reservoirs	X18	+	China Statistical Yearbook
		Total reservoir capacity	X19	+	China Statistical Yearbook
		Permanent population at the end of the year	X20	-	China Statistical Yearbook
	Industrial dimension	Proportion of secondary industry	X21	-	China Statistical Yearbook
		Proportion of tertiary industry	X22	+	China Statistical Yearbook
		Total agricultural output value	X23	+	China Statistical Yearbook
		Total output value of forestry	X24	+	China Statistical Yearbook
		Total output value of animal husbandry	X25	-	China Statistical Yearbook
	Energy consumption	Coal consumption	X26	-	China Statistical Yearbook
		Coke consumption	X27	-	China Statistical Yearbook
		Crude oil consumption	X28	-	China Statistical Yearbook
		Gasoline consumption	X29	-	China Statistical Yearbook
		Kerosene consumption	X30	-	China Statistical Yearbook
		Diesel consumption	X31	-	China Statistical Yearbook
		Fuel oil consumption	X32	-	China Statistical Yearbook
		Natural gas consumption	X33	-	China Statistical Yearbook
		Power consumption	X34	-	China Statistical Yearbook
	Economy and technology	Carbon decoupling index	X35	+	Tapia method calculation
		Green finance	X36	+	Yin Zibo et al.(2021)
		Digital economy	X37	+	Zhang Zeping et al.(2015)
		Number of green patent applications	X38	+	China Statistical Yearbook

#### 3.2.3 Use the entropy weight method to calculate the high-quality economic development index and the carbon neutral development index

In this paper, the entropy method is used to construct the indicators of high-quality economic development and carbon neutral comprehensive development. The specific steps are as follows:

First, select indicator *X*_*ij*_, where *i* is the region, *i* = 1,2,……*n*,*j* is the selected index, *j* = 1,2,……,*m*.

Second, the dimensionless treatment of indicators is carried out to eliminate the impact of different measurement units. The method is to take the maximum value and minimum value as the endpoints, and carry out linear processing. The result of dimensionless processing is between 0 and 1. The indicators are divided into positive indicators and negative indicators. The positive indicator means that the higher the indicator value is, the higher the score will be. The negative indicator means that the higher the indicator value is, the lower the score will be. See Formulas (6) and (7) for details:

$$Y_{ij}=\frac{X_{ij}-X_{min}}{X_{max}-X_{min}}$$ (6)

$$Y_{ij}=\frac{X_{max}-X_{ij}}{X_{max}-X_{min}}$$ (7)

Where, *X*_*max*_ and *X*_*min*_ represents the maximum and minimum values of an index, *Y*_*ij*_ and *X*_*ij*_ is a dimensionless index.

Determine the information entropy third. The system’s degree of disorder is gauged by the information entropy. While the two have the same absolute values, their signs are the exact opposite. Information is more disordered and has a lower utility value when the information entropy is higher. The opposite is also true. The precise formula is displayed in (8).


$$e_{ij}=-k\sum_{i=1}^{n}Y_{ij}lnY_{ij},k>0$$ (8)

$Y_{ij}=\frac{r_{ij}}{\sum_{i=1}^{n}r_{ij}}$ representing the proportion of index value of the *i* region under the *j* index

Fourth, calculate the difference coefficient *d*_*j*_. See Formula (9) for details.


$$d_{j}=1-e_{j}$$ (9)

Fifth, the weight of each indicator is obtained by normalizing the difference coefficient. The specific algorithm is shown in Formula (10).


$$W_{j}=\frac{d_{j}}{\sum_{i=1}^{n}d_{j}}$$ (10)

Sixth, calculate the comprehensive elderly care capacity indicators of each region. The specific algorithm is shown in Formula (11):

$$Y_{ij}=\sum_{j=1}^{n}W_{j}e_{ij}$$ (11)

#### 3.2.4 Mediating variable

Total factor productivity (*tfp*): tfp is an important indicator in the decomposition of economic growth sources, which is mainly reflected in a certain proportion between output and input in economic growth. This paper uses the Malmquist index method to calculate the total factor productivity of 30 provinces (municipalities directly under the Central Government) in China from 2011 to 2020 [31].

While the improvement of total factor productivity (TFP) is typically associated with economic growth and efficiency enhancement, it does not equate to economic high-quality development. Economic high-quality development is a more comprehensive and multidimensional concept that goes beyond the improvement of productivity, taking into account factors such as environmental sustainability, social equity, social welfare, and quality of life. The improvement of TFP may lead to economic growth, but it does not guarantee sustainable growth that benefits social welfare or enhances people’s quality of life. Therefore, it is necessary to study the impact of TFP on economic high-quality development. By delving into the changes in TFP and its effects on the economy, we can better understand the sources and drivers of economic growth, evaluate the effectiveness of policy measures, and provide recommendations for promoting economic high-quality development. Additionally, by considering the relationship between TFP and other factors such as the environment, society, and quality of life, we can comprehensively assess the overall effects and sustainability of economic development. This research can help us gain a better understanding of the overall picture of economic development and formulate more comprehensive, sustainable, and welfare-enhancing development strategies and policies [38].

#### 3.2.5 Control variable

1. Environmental regulation (*eri*): the environmental regulation index is used by regulatory authorities to guide the establishment and implementation of enterprises, reduce pollutant emissions, and achieve environmental protection and first-class economic development. In this study, the emissions of contemporary wastewater, contemporary sulfur dioxide, and contemporary residue are utilized to calculate the whole index of regional environmental indicators [39].
2. Economic openness (*open*): According to Yang et al. (2021), economic openness is determined by the proportion of each province’s total import and export commerce to its GDP for that year [40].
3. Unemployment rate (*ur*): According to Zhao et al. (2023) it refers to the officially declared unemployment rate, which may show how much of a country’s labor force is employed [41].
4. Foreign direct investment (*lnfdi*): The volume of foreign direct investment is used to gauge it [42].
5. PM2.5 concentration (*lnpm*): The main component of haze is PM2.5, and to some extent, the concentration of PM2.5 might indicate how bad the haze is. Provincial PM2.5 concentration data are obtained from the CSMAR database (China Stock Market & Accounting Research Database).
6. Urban-rural income gap (*gap*): It measures economic disparity by comparing urban and rural people’ disposable income [43].

### 3.3 Descriptive statistics

This paper employs Stata to generate descriptive statistics for the relevant model variables. Core explained variable: economic development of high quality (*ggdpsq*). Table 3 reveals that the maximum value is 0.896 while the minimum value is -0.038. The difference between the utmost value and the minimum value is substantial. China’s regional development is more heterogeneous, and there is a large disparity between the economically high-quality developed regions and the relatively underdeveloped regions. A standard deviation of 0.135 indicates that the dispersion of observations of high-quality economic development relative to the mean is small and the data points are relatively concentrated. Core explanatory variables (*score*): the maximum value of the carbon neutral development index is 0.591, the minimum value is 0.338 and the average value is 0.504, indicating that there are differences in carbon neutral development among regions in China, but the gap is relatively small. The details of other variables are shown in Table 3, and the original data of the variables in this paper are all from China Statistical Yearbook, China Financial Yearbook, China Environmental Statistical Yearbook, China Industrial Statistical Yearbook, China Insurance Yearbook, China Environmental Statistical Yearbook, Statistical Yearbooks of All Provinces, CSMAR database and Wind database. In order to ensure the scientific accuracy of empirical analysis and reduce the impact of variable heteroscedasticity, this paper deals with foreign direct investment, PM2.5 concentration and other variables by logarithmic method.

**Table 3:** Descriptive statistics.

Variable	Obs	Mean	SD	Min	Max
ggdpsq	300	0.253	0.135	-0.038	0.896
score	300	0.504	0.048	0.338	0.591
tfp	300	1.673	0.754	0.334	2.900
eri	300	0.532	0.553	-0.140	2.760
open	300	0.248	0.265	0.008	1.435
ur	300	3.207	0.665	1.200	4.500
lnfdi	300	-8.092	0.310	-8.785	-7.143
lnpm	300	3.601	0.391	2.120	4.451
gap	300	1.629	0.973	0.000	0.483

## 4. Results and discussion

### 4.1 Data stability test

Before regression, this paper conducts data stationarity test on the data, including unit root test, autocorrelation test, heteroscedasticity test, cross section correlation test and panel cointegration test. The results are shown in Tables 4–6:

**Table 4:** Unit root test.

**Unit root test**		**ggdpsq**	**score**	**eri**	**open**	**ur**
FISHER	Inverse chi-squared	258.760***	73.766	115.566***	128.382***	46.662
	Inverse normal	-9.420***	2.065	-1.216	-1.516*	3.448
	Inverse logit t	-11.983 ***	1.381	-2.097**	-2.905**	3.466
	Modified inv. chi-squared	18.144***	1.257	5.072***	6.242***	-1.218
LLC	Adjusted t	-23.680 ***	-9.003***	-3.763***	-7.619***	-3.145***
IPS	W-t-bar	-7.519**	0.470	1.945	-0.172	4.130
HADRI	z	18.527***	15.927 ***	18.443***	15.907***	14.031***
**Unit root test**		**lnfdi**	**lnpm**	**gap**	**tfp**	**urbanization**
	Inverse chi-squared	50.065	214.710***	2162.619***	235.558***	176.747***
FISHER	Inverse normal	3.540	-4.726***	-44.507***	-9.040***	0.301
	Inverse logit t	3.541	-8.662***	-109.200***	-11.281***	-3.379***
	Modified inv. chi-squared	-0.907	14.123***	191.942***	16.026***	10.658***
LLC	Adjusted t	-5.436***	-7.514***	0.980	-7.332***	-7.675***
IPS	z	1.527	0.636	0.750	0.957	-0.703
HADRI	W-t-bar	14.564 ***	15.855***	14.908***	17.481***	15.124***

Standard errors in parentheses

* p<0.1

** p<0.05

*** p<0.01.

**Table 5:** Unit root test autocorrelation, cross section, heteroscedasticity and panel cointegration tests.

Autocorrelation, cross section, heteroscedasticity and panel cointegration tests		statistic	p-value
Autocorrelation	**(**xtserial)	121.327	0.0000
Cross section correlation	pesaran	16.165	0.0001
Heteroscedasticity	\	194.99	0.0000
Pedroni	Modified Phillips-Perron t	9.6213	0.0000
Pedroni	Phillips-Perron t	-19.2471	0.0000
Pedroni	Augmented Dickey-Fuller t	-17.5759	0.0000
Westerlund	Variance ratio	409.5634	0.0000

**Table 6:** Results of FGLS test, PCSE test and variance expansion factor test.

	OLS	PCSE	FGLS	VIF
score	0.833***	0.833***	0.833***	2.040
	(0.165)	(0.079)	(0.162)	
eri	-0.002	-0.002	-0.002	2.420
	(0.016)	(0.011)	(0.016)	
open	0.025	0.025**	0.025	1.350
	(0.024)	(0.012)	(0.024)	
ur	-0.026***	-0.026***	-0.026***	1.320
	(0.010)	(0.006)	(0.009)	
lnfdi	0.180***	0.180***	0.180***	2.230
	(0.026)	(0.026)	(0.026)	
lnpm	0.180***	0.180***	0.180***	1.300
	(0.016)	(0.014)	(0.016)	
gap	0.008	0.008	0.008	1.520
	(0.007)	(0.010)	(0.007)	
_cons	0.710***	0.710***	0.710***	
	(0.211)	(0.151)	(0.208)	
Mean Vif				1.740

Standard errors in parentheses

* p<0.1

** p<0.05

*** p<0.01.

1. Each variable has passed at least two of FISHER, LLC, IPS and HADRI unit root tests, so the selected variable is reasonable, as show in Table 4.
2. The data has autocorrelation, heteroscedasticity, and cross section correlation, as show in Table 4, so FGLS and PCSE methods are modified for such cases. The results are shown in Table 6: Model (1a) OLS, Model (1b) PCSE and Model (1c) FGLS. The regression results of PCSE and FGLS are consistent with the structural basis of the benchmark regression.
3. The panel data passed the Pedroni and Westerlund cointegration tests, demonstrating the stability of the regression residuals and the existence of a long-term stable equilibrium relationship between the variables. The results of the regression are more accurate at this point because the original equation can later be directly regressed on this basis, as show in Table 5.
4. In order to verify whether there are multiple collinearities among variables, this paper conducts variance inflation factor test. The results are shown in Table 6. The average VIF value of each variable is 2.150, and the VIF value of a single variable is not greater than 5. Therefore, it is proved that there is no serious collinearity between variables, and subsequent model construction can be carried out.

### 4.2 Benchmark regression and spatial Durbin regression

Before building the spatial model, this paper has solved the global Moran index of the economic high-quality development (*ggdpsq*) and carbon neutral development index (*score*) from 2011 to 2020. The spatial matrix uses the geographical distance matrix, and the results are shown in Table 7. According to Table 7: From 2011 to 2020, the Moran index of high-quality economic development was significant at the significance level of 1%. The Moran index of the carbon neutral development index was significant at the significance level of 5% or 10%, except that it was not significant in 2012. This proves that there is a strong spatial autocorrelation, so it is reasonable to build a spatial model for research.

**Table 7:** Moran I test results.

Year	ggdpsq	score
2011	0.312***	0.161**
	(0.081)	(0.095)
2012	0.305***	0.111
	(0.080)	(0.095)
2013	0.253 ***	0.143*
	(0.084)	(0.095)
2014	0.248 ***	0.136*
	(0.079)	(0.095)
2015	0.336 ***	0.124*
	(0.076)	(0.094)
2016	0.343***	0.153**
	(0.075)	(0.093)
2017	0.080	0.173 **
	(0.074)	(0.093)
2018	0.186***	0.180**
	(0.081)	(0.093)
2019	0.272 ***	0.181**
	(0.078)	(0.093)
2020	0.329***	(0.179) **
	(0.079)	(0.093)

Standard errors in parentheses

* p<0.1

** p<0.05

*** p<0.01.

**Fig 1:**
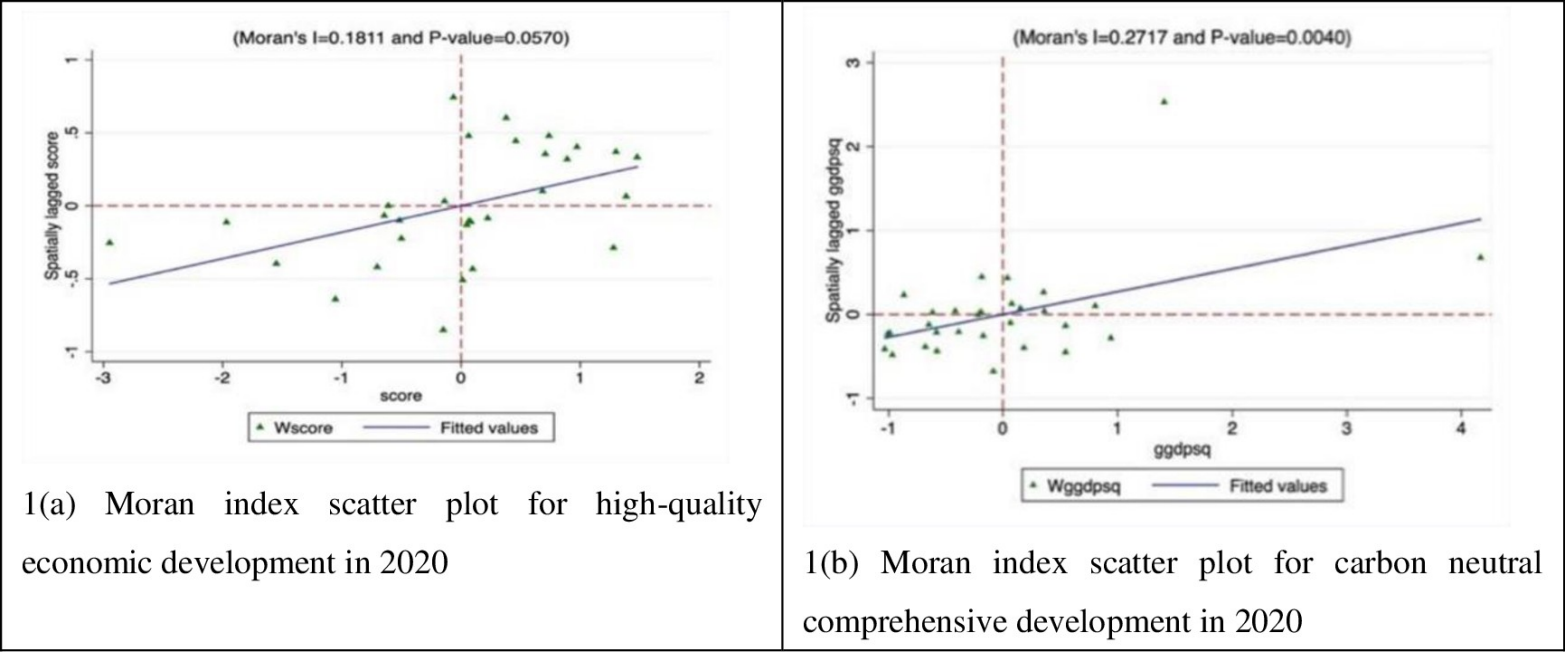
Moran’s I scatter plot in 2020. (a) Moran index scatter plot for high-quality economic development in 2020. (b) Moran index scatter plot for carbon neutral comprehensive development in 2020.

Fig 1’s Moran Scatter (1a) of high-quality economic development (*ggdpsq*) in 2020 shows that 8 provinces (26.67%) fall in the first quadrant and 12 provinces (40%) fall in the third, demonstrating that high-quality economic development has a positive spatial autocorrelation; The 2020 carbon neutral comprehensive development index (*score*) Moran’s Scatter (1b) reveals that 11 provinces (or 36.67% of the total) fall in the first quadrant and 10 provinces (or 33.33%) fall in the third, demonstrating that the index also exhibits a positive spatial autocorrelation.

#### 4.2.1 OLS panel regression and spatial regression

Before analyzing the regression results, this article first explains the goodness of fit (R). As shown in the regression results, the goodness of fit of OLS regression results is 0.620, which has a relatively good fit. Therefore, the regression results can be analyzed. At the 1% level of significance, Table 9 Model (1a) (*ols*) shows a significant positive correlation between the high-quality economic development (*ggdpsq*) and the carbon neutral comprehensive development index (*score*), with a coefficient of 0.833, indicating that carbon neutral development will significantly encourage high-quality economic development. The development of carbon neutrality can promote high-quality economic development from multiple perspectives such as high-quality, efficient, sustainable and improving people’s livelihood. For example, the reduction of extreme weather can improve the living environment of residents; The realization and advancement of low-carbon technology can be considerably aided by the establishment of carbon neutrality. Low-carbon technology innovation may enhance efficiency and quality at the same time, resulting in high-quality, efficient, and sustainable economic development. Therefore, Hypothesis 1(a) is confirmed. Based on the theory of innovation, carbon neutrality development encourages businesses and industries to seek more environmentally friendly and sustainable technologies and solutions. This kind of innovation drives technological progress, improves production efficiency, and enhances resource utilization efficiency. More efficient production methods can reduce energy consumption and lower carbon emissions, thereby promoting high-quality economic development. The empirical findings also indicate that foreign direct investment (*lnfdi*) both have a positive association at a significant level of 1%, indicating that they can both aid in the advancement of high-quality economic growth. Foreign direct investment will not only directly drive the economic growth of the investing countries, but also increase the human capital stock of the investing countries, thus indirectly promoting the national economic development. Foreign direct investment can accelerate the international flow of goods and factors, promote technological advancement, accelerate the accumulation of human resources and institutional mechanism innovation, continuously improve labor productivity, and improve the efficiency of resource allocation. By doing so, we can encourage high-quality domestic economic growth, particularly in the eastern region. Based on the theory of capital accumulation, FDI can attract a substantial inflow of capital, stimulating capital accumulation and investment growth. These funds can be utilized for infrastructure development, enhancing production capacity and technological levels, thereby leading to improved productivity and economic growth rates. With a coefficient of -0.026 and a significance level of 1%, we can also observe that the unemployment rate (*ur*) and qualitative economic development have a significant negative relationship. In other words, a declining unemployment rate supports promoting high-quality economic development. The unemployment rate is a key indicator of a nation’s economic development. The decline of the unemployment rate indicates that the economic development is getting better. More individuals are able to work at the occupations they prefer, which is good for both general economic growth and high-quality economic growth. Based on the Phillips curve theory, there exists a negative correlation between the unemployment rate and the inflation rate. When the economy experiences rapid growth and employment opportunities increase, the labor market becomes tight, leading to a decrease in the unemployment rate and an increase in wage levels. As wages rise, it increases the costs for businesses, which may restrain their investment and expansion plans, thereby impeding high-quality economic development.

The progress of carbon neutrality in the region will have an impact on the economic growth and prosperity of the surrounding areas. This study utilizes a spatial Durbin model regression to examine the geographical spillover effects of carbon neutrality development on high-quality economic growth. Before conducting the spatial Durbin regression, a series of experiments were performed, and the results are shown in Table 8. Firstly, the Wald test and LR test were conducted to determine the type of spatial regression model to use. The results indicate that both the Wald test and LR test are significant at a 1% level of significance. Therefore, the selected spatial Durbin model cannot be degraded into SEM or SAR models, making the choice of using the spatial Durbin model scientifically reasonable. Secondly, the Hausman test was conducted to determine whether to use a random effects model or a fixed effects model. The result is significant at a 1% level, with a coefficient of 16.230. Hence, the fixed effects model was chosen for regression. To further determine the type of fixed effects model to select, tests were conducted for time fixed effects, individual fixed effects, and double fixed effects options. The regression results for individual fixed effects and time fixed effects are significant at a 1% level, with coefficients of 64.680 and 337.260, respectively.

**Table 8:** Spatial model selection test.

Test	Value
lrtest both ind	64.68***
lrtest both time	337.26***
Hausman	16.230**
Wald_lag	43.63***
Wald_error	44.27***
LR_lag	41.26***
LR_error	42.94***

Standard errors in parentheses

* p<0.1

** p<0.05

*** p<0.01.

As shown in Table 9 Model (2), the direct effect (Main) in the regression results of the spatial Durbin model is generally consistent with the OLS regression results, indicating that carbon-neutral development can promote high-quality economic development. However, from the perspective of the spatial spillover effect, the relationship between the two is opposite, indicating that carbon-neutral development in this region is not conducive to high-quality economic development in adjacent regions. The reason why such a situation occurs and what causes it need to be further explored. Models (2a) (Main) and Models (2b) (Mx) show that the spatial spillover impact of carbon neutral development on high-quality economic growth is considerably negatively associated at the level of 1%, with a coefficient of -1.883. At the 10% level, there is a considerable positive correlation between the direct impact of carbon-neutral development and high-quality economic development, with a coefficient of 0.489. In other words, while carbon neutral development in this area can support high-quality economic growth there, it does not support such growth in the nearby areas. Therefore, Hypothesis 1(b) is confirmed.

**Table 9:** Results of OLS panel regression and Spatial regression.

	Model (1a) OLS	Model (2a) (Main)	Model (2b) (Wx)
score	0.833***	0.489**	-1.883***
	(0.165)	(0.212)	(0.675)
eri	-0.002	-0.001	-0.002
	(0.016)	(0.012)	(0.042)
open	0.025	-0.016	-0.279**
	(0.024)	(0.039)	(0.124)
ur	-0.026***	0.001	-0.049**
	(0.010)	(0.008)	(0.023)
lnfdi	0.180***	0.035	0.256***
	(0.026)	(0.025)	(0.054)
lnpm	0.180***	-0.068*	0.091
	(0.016)	(0.040)	(0.089)
gap	0.008	0.043***	0.001
	(0.007)	(0.013)	(0.026)
_cons	0.710***	Log-likelihood	564.666
	(0.211)	rho	0.251*
Hausman	40.92***		(0.088)
ID	YES		
YEAR	YES		
R^2^	0.620		
Obs	300		
Province	30		

Standard errors in parentheses

* p<0.1

** p<0.05

*** p<0.01.

So why do the direct effect and the spatial spillover effect have opposite results? There could be several reasons for this: Firstly, carbon-neutral development may lead to economic decline and job losses in certain regions or industries. During the process of carbon-neutral development, traditional high-carbon industries may face challenges as they need to undergo structural adjustments and transformations to meet the requirements of a low-carbon economy. This could result in reduced economic activities and job opportunities in these regions or industries, bringing negative impacts to the respective areas and labor markets. This spatial spillover effect suggests that carbon-neutral development may have adverse economic consequences in certain regions or industries. Secondly, carbon-neutral development may trigger resource reallocation and industrial restructuring. To achieve carbon neutrality goals, there is a need to reduce reliance on high-carbon energy and increase demand for low-carbon energy and clean technologies. This could lead to a reallocation of resources from traditional high-carbon industries to low-carbon industries, thereby adversely affecting some regions and companies. Such resource reallocation and industrial restructuring may result in reduced economic activities or impacts on certain regions and companies, generating negative spatial spillover effects. Additionally, carbon-neutral development may increase costs for businesses, especially those in high-carbon emission industries. Carbon pricing mechanisms, such as carbon emission trading systems or carbon taxes, may raise production costs for companies. This could have negative effects on their profitability and competitiveness, thereby impacting high-quality economic development.

#### 4.2.2 Regression results and analysis of mediating effect model

As shown in Table 10, the relationship between carbon neutral development and high-quality economic development is statistically significant at the significance level of 1%, with a coefficient of 0.833 indicating a significant contribution of carbon neutral development to high-quality economic development. This effect is shown in Path (1) and is shown in Table 9. Since carbon neutral development can significantly increase total factor productivity, Hypothesis 2 is confirmed. Path (2) regression results indicate that carbon neutral development has a significant impact on total factor productivity at the 1% significance level, with a coefficient of 2.980. This result can be explained by the following theories. Firstly, based on the theory of environmental economics, carbon-neutral development requires the implementation of carbon pricing mechanisms, such as carbon emission trading systems or carbon taxes. These mechanisms increase the production costs for businesses and incentivize them to adopt measures to reduce carbon emissions. By reducing waste and improving resource utilization efficiency, businesses can lower carbon emissions while simultaneously enhancing production efficiency, thereby promoting the improvement of total factor productivity. Secondly, based on the theory of human capital, investment in human capital can enhance the productivity and creativity of workers. In the context of carbon-neutral development, cultivating talents with green technology skills and environmental awareness can drive the application and innovation of green technologies, leading to increased production efficiency and total factor productivity improvement. Path (3) demonstrates that raising total factor productivity can encourage the growth of high-quality economies, and it is significant at the 1% level of significance with a coefficient of 0.022. Therefore, hypothesis 3 is verified. This result can be explained by the Newman growth theory, which focuses on the impact of economic structure and resource allocation on economic growth. The improvement of total factor productivity can enhance resource allocation and optimize economic structure. By enhancing production efficiency and resource utilization efficiency, the improvement of total factor productivity can achieve more effective resource allocation, making the economy more efficient, flexible, and adaptable. This optimization of economic structure and improvement of resource allocation can promote high-quality economic development, including improving output quality, reducing production costs, enhancing competitiveness, and so on.

**Table 10:** Results of mediating effect regression analysis.

	Path (1)	Path (2)	Path (3)
Variable	ggdpsq	tfp	ggdpsq
tfp	-	-	0.022***
			(0.008)
score	0.833***	2.980**	0.767***
	(0.165)	(1.217)	(0.164)
eri	-0.002	0.279**	-0.008
	(0.016)	(0.115)	(0.015)
open	0.025	0.069	0.023
	(0.024)	(0.178)	(0.024)
ur	-0.026***	0.079	-0.042***
	(0.010)	(0.070)	(0.009)
lnfdi	0.180***	0.410**	0.171***
	(0.026)	(0.196)	(0.026)
lnpm	0.180***	-0.235**	0.185***
	(0.016)	(0.119)	(0.160)
gap	0.008	-0.329***	0.015**
	(0.007)	(0.052)	(0.007)
_cons	0.710***	4.453***	0.612***
	(0.211)	(1.562)	(0.212)
R^2^	0.620	0.518	0.634
Obs	300		
province	30		

Standard errors in parentheses

* p<0.1

** p<0.05

*** p<0.01.

The condition for the independent variable’s effect on the dependent variable to be equal to zero or significantly reduced after controlling for the mediator variable is that the mediating effect is satisfied. Table 10 regression results show that the overall effects of the carbon-neutral development path (1) on promoting high-quality economic growth are 0.833 and 0.767, respectively, which are more significant than the direct effects of path (3). The direct effect distinguishes the impact of total factor productivity on high-quality economic growth, while the indirect effect incorporates the indirect effect of total factor productivity into the overall effect. Therefore, the coefficient of the direct effect is higher than that of the indirect effect. This result satisfies the definition of partial mediating effect, indicating that total factor productivity plays a partial mediating role between carbon-neutral development and high-quality economic development.

The empirical results show that the development of carbon neutrality can improve total factor productivity, which in turn will promote high-quality economic development. Through government regulations, green financing, green innovation, and other methods, the creation of carbon neutrality may spur technical advancement and increase the effectiveness of resource allocation. On the one hand, it can encourage workers to transition from low-productivity industries to high-productivity ones, which will encourage industrial structure optimization and upgrading and, in turn, encourage rational and efficient economic development; on the other hand, it denotes that the level of productive technology has advanced. In order to promote the transformation of the industry from a low value-added traditional industry to a high value-added high-tech, strategic emerging industry, the industry will be driven to develop in the direction of knowledge intensification, improve the knowledge intensity of the entire industry, and then improve the intensity of economic growth in order to achieve high-quality economic development.

This study uses the mediating effect model of regression analysis to examine the mediation effect of total factor productivity on the impact of carbon neutrality goal realization on high-quality economic development. Since the traditional three-step method omits endogenous variables in the regression, this paper conducts a self-sampling method with 500 iterations to test the mediating effect. The results are shown in Table 11, where _*bs*_1 represents indirect effect impact _*bs*_2 represents direct effect impact. The results show that the coefficient of _*bs*_1 is 0.066, and it is significant at the 10% significance level, which proves that through the mediation of total factor productivity, carbon neutrality development can promote high-quality economic development, and there is a significant mediating role. In addition, this paper also uses the concept of mediation variable to verify whether there is a mediating effect. (1) The change in independent variables can significantly explain the change in dependent variables; (2) The change in independent variables can significantly explain the change in intermediary variables; (3) After controlling for the mediating variable, the influence of the independent variable on the dependent variable should be equal to 0 or significantly reduced, while the mediating variable has a significant impact on the dependent variable. As shown in Table 10, path (1), carbon neutrality development significantly promotes high-quality economic development, meeting the conditions that changes in independent variables can significantly explain changes in dependent variables; As shown in the path (2) of Table 10, carbon neutrality development has significantly promoted the improvement of total factor productivity, meeting the conditions that independent variable changes can significantly explain changes in intermediary variables; As shown in Table 10, Path (3), after controlling the intermediate variable (*tfp*), the role of carbon neutrality development in promoting high-quality economic development has changed from 0.807 to 0.767, and the improvement of total factor productivity has a significant role in promoting high-quality economic development. After satisfying the control of the intermediate variable, the influence of the independent variable on the dependent variable should be equal to 0 or significantly reduced. Meanwhile, the significance of the mediating variable affects the conditions of the dependent variable. Therefore, the regression of mediating effect in this paper is scientific and stable.

**Table 11:** Bootstrap sampling regression results.

Sobel	0.066 * (0.036)
Goodman-1 (Aroian)	0.066 * (0.037)
Goodman-2	0.066 * (0.034)
_bs_1	0.066 * (0.040)
_bs_2	0.767 *** (0.164)

Standard errors in parentheses

* p<0.1

** p<0.05

*** p<0.01.

### 4.3 Discussion on robustness test result

#### 4.3.1 Robustness test of benchmark regression

To validate the robustness of the results, this study employs a one-period lag in the explanatory variables and control variables to examine endogeneity issues. The results, as shown in Table 12 (1), are consistent with the OLS regression results, indicating that the model used in this study does not suffer from severe endogeneity problems. In addition, this study conducts robustness tests using two techniques: expanded control variables and two-stage least squares (2SLS). The regression results, as shown in Table 12 (2) and (3), are also consistent with the OLS results, demonstrating the robustness and scientific validity of the regression model constructed in this study.

**Table 12:** Robustness test of benchmark regression.

(1)		(2)		(3)	
score1	0.840***	score	0.850***	score	0.655***
	(0.178)		(0.153)		(0.182)
eri1	0.002	eri	0.010	eri	-0.023
	(0.017)		(0.015)		(0.017)
open1	0.015	open	-0.092***	open	-0.0126
	(0.025)		(0.028)		(0.029)
ur1	-0.027**	ur	-0.031***	ur	-0.019*
	(0.010)		(0.010)		(0.010)
lnfdi1	0.200***	lnfdi	0.138***	lnfdi	0.129***
	(0.029)		(0.026)		(0.030)
lnpm1	0.186***	lnpm	0.160***	lnpm	0.196***
	(0.017)		(0.015)		(0.017)
gap1	0.005	gap	-0.016**	gap	0.086***
	(0.008)		(0.007)		(0.014)
-		urbanization	0.005**	-	-
-			(0.001)	-	-
cons	0.852***	cons	0.236	cons	0.156
	(0.229)		(0.209)		(0.239)
Obs	270	Obs	300	Obs	240
R^2^	0.530	R^2^	0.579	R^2^	0.593

Standard errors in parentheses

* p<0.1

** p<0.05

*** p<0.01.

#### 4.3.2 Robustness test of spatial Durbin regression

In this study, the 0–1 adjacent spatial weight matrix and economic distance matrix were used to replace the spatial geographic distance weight matrix. Table 13 shows the results. The results indicate that the regression results of the 0–1 matrix and economic distance matrix of the core explanatory variables are basically consistent with the regression conclusions of the spatial geographic distance weight matrix, thus inferring that the empirical results are reliable.

**Table 13:** Decomposition of spatial effects based on national sample.

	Model (distance)		Model (0–1)		Model (economics)	
	MAIN	MAIN	WX	WX	MAIN	WX
score	0.489**	-1.883***	0.542**	-0.864*	0.460*	-0.182*
	(0.212)	(0.675)	(0.227)	(0.445)	(0.251)	(0.466)
eri	-0.001	-0.002	0.010	-0.019	-0.012	-0.018
	(0.012)	(0.042)	(0.012)	(0.022)	(0.015)	(0.026)
open	-0.016	-0.279**	-0.044	0.019	0.035	-0.199*
	(0.039)	(0.124)	(0.042)	(0.056)	(0.044)	(0.114)
ur	0.001	-0.049**	-0.002	0.024	0.009	0.011
	(0.008)	(0.023)	(0.008)	(0.019)	(0.010)	(0.023)
lnfdi	0.035	0.256***	0.020	0.151***	0.044	-0.028
	(0.025)	(0.054)	(0.027)	(0.047)	(0.032)	(0.065)
lnpm	-0.068*	0.091	-0.027	0.029	0.059	-0.022
	(0.040)	(0.089)	(0.044)	(0.070)	(0.038)	(0.063)
gap	0.043***	0.001	0.039***	0.0022	0.013	-0.009
	(0.013)	(0.026)	(0.013)	(0.027)	(0.018)	(0.020)
Obs	300					
Province	30					

Standard errors in parentheses

* p<0.1

** p<0.05

*** p<0.01.

### 4.4 Analysis of regional heterogeneity

To further investigate the impact of carbon-neutral development on high-quality economic development in China, this study divides the country into three regions: the Eastern, Central, and Western regions, for the purpose of conducting regional heterogeneity analysis. There are several reasons for conducting this analysis: Firstly, China has vast geographical diversity and regional disparities in natural endowments and economic development. The Eastern, Central, and Western regions of China exhibit significant differences in natural environment, economic structure, and development levels. The Eastern region is relatively developed with a more diversified economic structure, while the Central and Western regions are relatively less developed with relatively homogeneous economic structures. By dividing the regions into Eastern, Central, and Western, we can better identify and analyze the regional differences, thereby gaining deeper insights into the relationship between carbon-neutral development and high-quality economic development. Secondly, this analysis enables more targeted policy-making. The Eastern, Central, and Western regions face different challenges and opportunities in terms of carbon-neutral development and high-quality economic development. By conducting regional heterogeneity analysis, we can provide more targeted recommendations and measures for policy-making. Developing policies and measures tailored to the characteristics and needs of different regions can facilitate the implementation of carbon-neutral development and high-quality economic development. Lastly, it facilitates the formation of a knowledge-sharing system. Dividing China into Eastern, Central, and Western regions helps promote knowledge sharing and experience exchange among different regions. Each region can learn from the successful experiences and best practices of other regions in carbon-neutral development and high-quality economic development. This knowledge sharing contributes to improving the overall level of carbon-neutral development and high-quality economic development across the country.

The regression results, as shown in Table 14, indicate that carbon neutrality development has a significant positive impact on high-quality economic development in both the eastern and central regions, with coefficients of 0.913 and 0.758 respectively, both significant at the 1% level. However, in the western region, although the coefficient remains positive, it is not significant. There are several reasons for the regional differences: (1) Economic structure: The eastern and central regions have relatively diversified economic structures, including manufacturing, services, and high-tech industries. These regions have made significant progress in industrial upgrading and transformation, making it easier to achieve high-quality economic development through adjusting industrial structures and promoting clean technology innovation. In contrast, the western region relies more on traditional resource-based industries, resulting in a relatively smaller impact of carbon neutrality development on its economic development. (2) Resource endowment: The eastern and central regions have richer economic resources and a stronger foundation for technological innovation compared to the western region. This makes it easier for the eastern and central regions to implement carbon neutrality development policies and measures, thus facilitating high-quality economic development. (3) Market demand: The eastern and central regions have larger market sizes and more diverse consumer demands. This provides broader market space and opportunities for promoting the development of carbon neutrality industries, further driving high-quality economic development. In contrast, the western region has a smaller market size and relatively limited consumer demand, which restricts the development of carbon neutrality industries and high-quality economic development. (4) Policy support: The eastern and central regions have received more policy support and investment in carbon neutrality development and high-quality economic development. The governments in these regions place a greater emphasis on environmental protection and sustainable development. They provide preferential policies, financial support, and technological innovation to promote carbon neutrality development and high-quality economic development. In contrast, the policy support in the western region is relatively limited, which hinders the progress of carbon neutrality development and high-quality economic development.

**Table 14:** Regional heterogeneity regression results.

	Eastern	Middle	Western
score	0.913***	0.758***	0.423
	(0.285)	(0.136)	(0.368)
eri	-0.016	-0.060***	-0.013
	(0.019)	(0.014)	(0.046)
open	0.351***	-0.961***	0.017
	(0.062)	(0.130)	(0.148)
ur	-0.031**	-0.037***	0.002
	(0.013)	(0.010)	(0.017)
lnfdi	0.073**	0.010	-0.059
	(0.033)	(0.023)	(0.086)
lnpm	0.072***	0.128***	0.018
	(0.021)	(0.017)	(0.027)
gap	0.162***	-0.032	0.143
	(0.019)	(0.021)	(0.147)
_cons	0.198	-0.293	0.612***
	(0.280)	(0.229)	(0.212)
ID	YES	YES	YES
YEAR	YES	YES	YES
Obs	110	100	90
province	11	10	9

Standard errors in parentheses

* p<0.1

** p<0.05

*** p<0.01.

## 5. Conclusion and policy implication

This study utilizes panel data from 30 provinces in mainland China from 2011 to 2020 for regression analysis and draws the following conclusions: (1) Carbon-neutral development has a positive main effect on economic high-quality growth, but there are negative spatial spillover effects. (2) Carbon-neutral development significantly improves total factor productivity (TFP), and the significant improvement in TFP promotes high-quality economic growth. (3) Carbon-neutral development has a significant promoting effect on economic high-quality development in the eastern and central regions of China, while it is not significant in the western region. The following recommendations are made in this report based on the findings:

1. Establish Carbon Neutrality Incentive Mechanisms: The government can formulate incentive policies such as carbon emission trading systems or carbon taxes to encourage companies to reduce carbon emissions. Additionally, rewards and preferential policies can be provided to companies that excel in carbon neutrality development, thereby promoting high-quality economic development.
2. Enhance Policy Support for Total Factor Productivity Improvement: Total factor productivity plays an intermediary role in promoting high-quality economic development through carbon neutrality. The government can strengthen policy support in relevant areas. For example, providing more research and development funding and innovation support to promote technological progress and innovation capabilities. Additionally, enhancing education and training to improve labor quality and skill levels can boost total factor productivity. These policies will contribute to further advancing carbon neutrality development and high-quality economic development.
3. Strengthen Cross-departmental Cooperation and Policy Coordination: The impact of carbon neutrality development on high-quality economic development varies across regions. The government should enhance the establishment of cross-departmental cooperation and policy coordination mechanisms to ensure the consistency and synergy between carbon neutrality development policies and high-quality economic development policies. Furthermore, the establishment of evaluation mechanisms and monitoring systems should be prioritized to enable timely policy adjustments and effective evaluation of policy outcomes. This will contribute to enhancing policy relevance and feasibility, further driving carbon neutrality development and high-quality economic development.
4. Formulate Regionally Differentiated Carbon Neutrality Development Policies: Considering the significant promotion of high-quality economic development through carbon neutrality in eastern and central regions of China, the government can further develop specific regionally differentiated policies. For instance, in the eastern and central regions, increasing support for clean energy development, encouraging green technology innovation, and facilitating industrial transformation can drive carbon neutrality development and high-quality economic development. In the western region, more targeted policies can be implemented, such as increased investment in green infrastructure construction and promotion of green industry development, to enhance the role of carbon neutrality development in driving economic growth.

## Supporting information

S1 Data (DTA)
